# Diverse bacterial genomes encode an operon of two genes, one of which is an unusual class-I release factor that potentially recognizes atypical mRNA signals other than normal stop codons

**DOI:** 10.1186/1745-6150-1-28

**Published:** 2006-09-13

**Authors:** Pavel V Baranov, Bente Vestergaard, Thomas Hamelryck, Raymond F Gesteland, Jens Nyborg, John F Atkins

**Affiliations:** 1 Bioscience Institute, University College Cork, Cork, Ireland; 2 Department of Human Genetics, University of Utah, 15N 2030E, Salt Lake City, UT84112-5330, USA; 3 Department of Molecular Biology, University of Aarhus, Gustav Wieds Vej 10C, DK-8000 Aarhus C, Denmark; 4 Department of Medicinal Chemistry, Danish University of Pharmaceutical Sciences, Universitetsparken 2, DK-2100 Copenhagen, Denmark; 5 Bioinformatics center, Institute of Molecular Biology and Physiology, University of Copenhagen, Universitetsparken 15, Building 10, 2100 Copenhagen, Denmark

## Abstract

**Background:**

While all codons that specify amino acids are universally recognized by tRNA molecules, codons signaling termination of translation are recognized by proteins known as class-I release factors (RF). In most eukaryotes and archaea a single RF accomplishes termination at all three stop codons. In most bacteria, there are two RFs with overlapping specificity, RF1 recognizes UA(A/G) and RF2 recognizes U(A/G)A.

**The hypothesis:**

First, we hypothesize that orthologues of the *E. coli *K12 pseudogene *prfH *encode a third class-I RF that we designate RFH. Second, it is likely that RFH responds to signals other than conventional stop codons. Supporting evidence comes from the following facts: (i) A number of bacterial genomes contain *prfH *orthologues with no discernable interruptions in their ORFs. (ii) RFH shares strong sequence similarity with other class-I bacterial RFs. (iii) RFH contains a highly conserved GGQ motif associated with peptidyl hydrolysis activity (iv) residues located in the areas supposedly interacting with mRNA and the ribosomal decoding center are highly conserved in RFH, but different from other RFs. RFH lacks the functional, but non-essential domain 1. Yet, RFH-encoding genes are invariably accompanied by a highly conserved gene of unknown function, which is absent in genomes that lack a gene for RFH. The accompanying gene is always located upstream of the RFH gene and with the same orientation. The proximity of the 3' end of the former with the 5' end of the RFH gene makes it likely that their expression is co-regulated *via *translational coupling. In summary, RFH has the characteristics expected for a class-I RF, but likely with different specificity than RF1 and RF2.

**Testing the hypothesis:**

The most puzzling question is which signals RFH recognizes to trigger its release function. Genetic swapping of RFH mRNA recognition components with its RF1 or RF2 counterparts may reveal the nature of RFH signals.

**Implications of the hypothesis:**

The hypothesis implies a greater versatility of release-factor like activity in the ribosomal A-site than previously appreciated. A closer study of RFH may provide insight into the evolution of the genetic code and of the translational machinery responsible for termination of translation.

**Reviewers:**

This article was reviewed by Daniel Wilson (nominated by Eugene Koonin), Warren Tate (nominated by Eugene Koonin), Yoshikazu Nakamura (nominated by Eugene Koonin) and Eugene Koonin.

## Open peer review

Reviewed by Daniel Wilson (nominated by Eugene Koonin), Warren Tate (nominated by Eugene Koonin), Yoshikazu Nakamura (nominated by Eugene Koonin) and Eugene Koonin. For the full reviews, please go to the Reviewers' comments section.

## Background

The synthesis of all mRNA-encoded proteins is performed by the ribosome. To decode mRNA, ribosomes use mediator molecules to link codon identity and meaning. For codons specifying amino acids, tRNA molecules serve as the mediators. Specific matching of codons and tRNAs is accomplished on ribosomes which select cognate tRNAs based on features of the geometry of the corresponding codon:anticodon duplexes [1]. In contrast, for codons that are signals for termination of translation, protein molecules serve the role as the mediators. These proteins recognize the three stop codons in mRNA and are termed class-I release factors (RFs) [2]. In most eukaryotes and archaea (except for special cases described below) there is a single RF responsible for termination at all three stop codons [3]. In most bacteria, there are two RFs with overlapping selectivity to stop codons [4,5]; RF1 recognizes UA(A/G) stop codons, and RF2 recognizes U(A/G)A stop codons.

**Figure 1:**
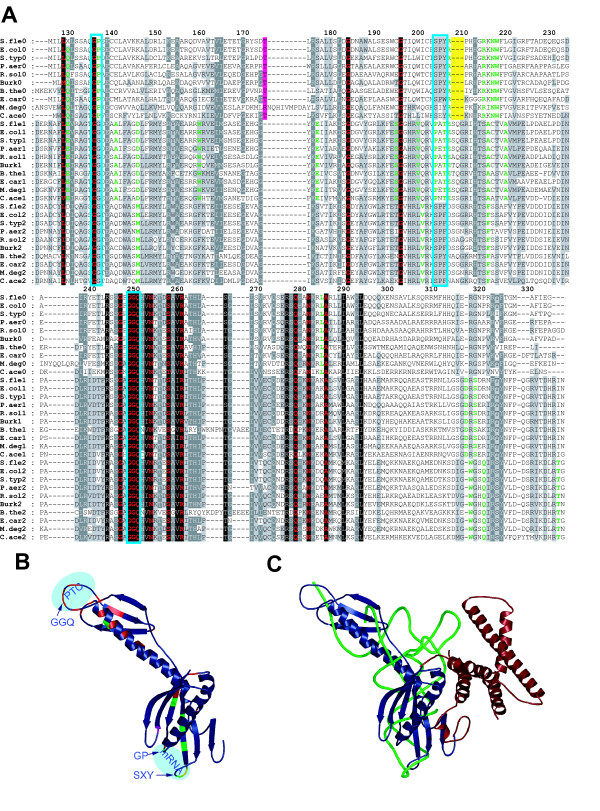
**Sequence comparison of release factors and structural model of RFH**. **A**. Multiple alignment of RF sequences from bacteria with three RFs numbered according to the *E. coli *RF2 sequence. The N- and C-termini of RF1 and RF2 are not present in RFH and are excluded from the alignment. Abbreviations for organisms and gene bank accession numbers for complete genomes are: *B.the *– *Bacteroides thetaiotaomicron *VPI-5482 [NC_004663]; *C.ace *– *Clostridium acetobutylicum *[NC_003030]; *Burk *(or *B.xen*) – *Burkholderia xenovorans *(JGI, see text); *E.car *– *Erwinia carotovora *[NC_004547]; *E.col *– *Escherichia coli *CFT073 [NC_004431]; *M.deg *– *Microbulbifer degradans *(JGI, see text); *P.aer *– *Pseudomonas aerugenosa *[NC_002516]; *R sol *– *Ralstonia solanacearum *[NC_003296]; *S.fle *– *Shigella flexneri *2a [NC_004337]; *S.typ *– *Salmonella typhi *CT18 [NC_003198]. Conserved residues are highlighted in color. The red color is used for those residues that are conserved in all three RF families. Green is used for residues that are specifically conserved for one type of factor, i.e. 100% conserved in RF1 and never appears in RFH or RF2. The remaining conserved residues are differentially shadowed in grey. The conserved deletion and insertion in RFH is marked in yellow and purple respectively. Boxes mark the occurrence of functionally important sequence motifs: the GG/GP motif contacting position one of the stop codon, the anticodon motifs and the GGQ-motif. Multiple alignment was produced using ClustalW [23]. **B**. Cartoon representation of the model of RFH colored as in panel A. The model was made using the program Modeller [44], with pdb-files 2B9M and 2B64 (chain Y) as structural models and the above alignment as input. The figure has been produced using PyMol [45]. Areas corresponding to the peptidyl transferase center (PTC) and mRNA positions are marked in light blue. The GP, GGQ and SXY motifs are marked with arrows. **C**. Cartoon representation of a superposition of the structural model of RFH (blue), A-site tRNA (green) and *Thermus thermophilus *RF2 (red). RF2 and tRNA are from pdb-files 2B9M and 1HIX. Only domains not present in RFH are shown from RF2 (residues 1–114 and 320–364, *T. term *numbering).

RF1 and RF2 share significant sequence and structural similarity [6-8]. The proteins are organized in four protein domains that play different functional roles [9]. Domain 3 contains a GGQ motif that is believed to be responsible for hydrolysis of the peptidyl bond during termination. The GGQ motif is the sole universally conserved motif in class-I RFs from all kingdoms of life [3,10]. Domains 2 and 4 together form a superdomain that is responsible for stop codon recognition in mRNA. This superdomain shares significant structural and sequence similarity between RF1 and RF2. Two Gly residues in the tip of the alpha 5 helix (boxed in Fig 1A) are thought to be in contact with the uridine in the first position of the stop codon exposed in the ribosomal A-site [9]. These two Gly residues are universally conserved in all bacterial RF1 and RF2 sequences [11]. There are specific conserved differences between RF1 and RF2 associated with different stop codon selectivity of these factors. Genetic studies demonstrated that these differences involve the PXT motif in RF1 and the SP(F/Y) motif situated in the corresponding position in RF2 [12,13]. Since these motifs can be compared to tRNA anticodons, they are sometimes referred to as the "RF anticodons". We will use this term further for simplicity. Biochemical data [14,15] followed by structural studies revealed that such "RF anticodons" are in close proximity (if not in direct contact) to positions 2 and 3 of stop codons [9,16-18]. Domain 1 is thought to bind to the class-II release factor RF3 (GTPase that promotes activity and recycling of class-I RFs [19]). This is the least conserved domain in RFs and it is differently oriented in RFs upon binding to the ribosome [9,16-18]. This domain is not essential for the function of RFs in stop codon recognition and peptidyl hydrolysis [13].

In 1992, Pel et al [20] identified an *E. coli *K12 genomic element encoding a protein sequence that shares significant similarity with RF1 and RF2 and named it *prfH *(*p*rotein *r*elease *f*actor *h*omologue). Here we analyzed the numerous bacterial genome sequences that have since become available and revealed that many bacteria encode *prfH *orthologs, which contain no discernable ORF interruptions. It has also become evident that the original *E. coli *K12 *prfH *gene was N-terminally truncated. To our knowledge, expression of the *prfH *gene in any bacteria has never been shown. Detailed analysis of protein sequences encoded by these genes and modeling a corresponding three-dimensional structure led us to the hypothesis that these genes encode a class-I RF that terminates protein synthesis at unknown signals. In this article, we describe supportive evidence for this hypothesis, its implication for a basic understanding of translation termination in bacteria and suggest experiments that will help to elucidate the particular function of the *prfH*-encoded protein that we further call RFH.

## The hypothesis

We have analyzed 311 completed bacterial genomes available at NCBI [21] on 20^th ^of May 2006 for the presence of Class-I RFs using ARFA program [22] Our analysis revealed that 23 of them contain either intact or disrupted ORFs encoding RFH. Figure 1 shows an alignment of RF1, RF2 and RFH sequences from representative bacteria that encode all three factors (Fig. 1A) and a structural model of RFH (Fig.1B) highlighting the differential conservation pattern between RFH, RF1 and RF2 (see figure legend for details). We provide an alignment of all release factors from analyzed bacteria in the nexus format [see Additional file 1]. Nucleotide sequences were extracted using custom designed perl scripts and ARFA program [22]. Protein sequences were aligned using ClustalW [23], then protein alignment was backtranslated to obtain codon alignment.

First, it is clear that all three factors share significant similarity in the area of the peptidyl hydrolysis domain including the GGQ motif (Fig. 1A). Due to the presence of this motif in RFH it is placed in the same cluster of orthologous groups (COG1186J) with RF1, RF2 and *yaeJ *(function is unknown) [24]. RFH shares similarity with other RFs throughout its entire sequence (in some genomes it is mistakenly annotated as RF2 [22]). *yaeJ *similarity is limited to GGQ motif and it is highly distinct from RFs in other areas of its sequence. More strikingly, RFH sequences from different bacteria have their most conserved residues in the areas corresponding to those known to have functional importance in class-I RFs. Most interestingly, the putative RFH "anticodon" is SXY which is somewhat similar to the RF2 anticodon SP(F/Y). In addition, the alignment contains a conserved gap of three amino acids corresponding to the RFH anticodon loop (shown in yellow on Fig. 1). In the area of contact of RFs with the first position of the stop codon (boxed in the alignment), RFH has a conserved GP sequence instead of the strictly conserved GG in RF1 and RF2. Finally, in RFH there is one additional amino acid in the loop around position 172 (*E. coli *RF2 numbering, purple in Fig. 1B). All together, these combined differences suggest different codon specificity for RFH. In addition, a substitution of negative Glu residues with positive residues in the area of the mRNA recognition domain changes specificity of RFs [25,26]. At least one such change is obvious at the position adjacent to the RF2 "anticodon" from the C-terminus. In RFH there is a universal positive Arg residue instead of the usually negative residue in RF1 and RF2.

The most dramatic difference between RFH and the other two factors is the lack of the N-terminal coiled coil domain 1 (Fig. 1B and 1C). This domain is the least conserved of the RF domains and it is in a different orientation in RF1 and in RF2 bound to ribosomes [9,18]. Studies of the *in vivo *and *in vitro *effect of swapping or deleting the N-terminal domain show that this domain has no effect on codon specificity, but is necessary to stimulate nucleotide exchange on the Class-II RF, RF3 [13]. It is noteworthy that the N-terminal domain is not necessary for *in vitro *peptide release, and that truncated RF1 functions *in vivo*, and has a similar conformation in solution [6]. It has been suggested by small-angle X-ray scattering analysis that domain 1 is flexible in solution [6]. This further adds to the impression that domain 1 is not an integrated part of the essential RF activity, but could have been added in the course of evolution for optimizing the process of peptide release, when RF3-mediated recycling, *via *the contact with domain 1, speed up the overall termination process. Moreover, the RF3 encoding gene, *prfC *is not essential in *E. coli *[27,28] and its orthologs have not been identified in bacteria with small genomes. Thus, despite the lack of domain 1, RFH could be a fully active class-I RF, capable of promoting peptidyl-tRNA hydrolysis and polypeptide chain release.

RFH resembles the shape of a tRNA molecule more closely than RF1 or RF2 (Fig. 1C), mostly due to the lack of domain 1. The close resemblance to the shape of a tRNA molecule further supports our hypothesis, that RFH has its natural active site in the ribosomal A-site, as other RFs.

In summary, RFH is very similar to other RFs in the area of the peptidyl hydrolysis domain that accomplishes the basic function in all class-I RFs. Additionally it shares significant similarity in the areas responsible for mRNA recognition, but contains a number of conserved changes specific to RFH, suggesting that its recognition properties differ from those of RF1 and RF2. The overall pattern of conservation within RFH is nearly the same as in RF1 and RF2 strongly suggesting that RFH functions as a class-I RF.

**Figure 2:**
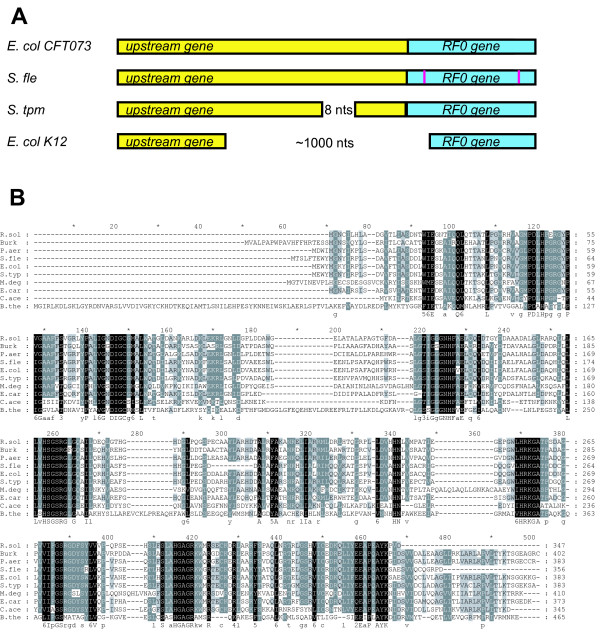
**Comparative schematic gene alignment of RFH operons and alignment of upstream gene**. **A**. Schematic representation of disrupted ORFs among the sequences analyzed. The RFH gene and its upstream companion are shown as boxes. Deleted regions are shown as lines and the sizes of deletions are indicated. Nonsense mutations are shown by vertical purple lines. **B**. Sequence alignment of the upstream gene produced with ClustalW [23]. For abbreviations and accession numbers see legend to Fig. 1.

Analysis of the sequence surrounding the RFH gene in different bacteria shows that in each observed case there is a specific gene 5' of the RFH gene (Fig. 2). These upstream genes share significant sequence similarity. Genes that share the same level of sequence similarity are absent in those bacteria that lack RFH. The putative proteins encoded by these upstream genes belong to a larger superfamily of RtcB-like proteins. Members of this superfamily are present in all kingdoms of life, but their functions are unknown [29], although it has been suggested that they are associated with tRNA or rRNA processing [30]. The crystal structure of archaeal RtcB was recently solved [31]. Unfortunately, its structure does not offer even faint inklings regarding RtcB function, though it revealed a novel protein fold. Translation of the RFH gene and its accompanying upstream gene is likely coupled, e.g. the stop codon of the 5' gene and the start codon for the RFH encoding sequence are always in very close proximity and their ORFs often overlap. Conserved co-regulation of translation suggests a relation between functions and conserved co-localization in bacteria [32,33] and points to physical interactions between the encoded products [34]. Perhaps the most obvious suggestion for the potential function of the product of the upstream gene is that it substitutes the missing domain 1. However, there is no detectable sequence similarity between RF domain 1 and the translation product of the upstream gene, nor any structural similarity to the protein fold of *rtcB*, and thus there is no apparent reason to believe that the upstream gene product has a function corresponding to the function of domain 1. Another speculative idea links a suggested function of the upstream gene with tRNA/mRNA processing to RF activity [30]. It is possible that a (specific) tRNA modifying enzyme would cause a codon specific translational stop, which could then be terminated by RFH action. Another option is that RFH releases stalled ribosomes, assisted by mRNA or perhaps even rRNA modifications by the upstream gene.

Hints regarding the RFH functional role potentially could be obtained from its evolutionary history. For example, if RFH were a progenitor of RF1 and RF2, it would be reasonable to expect that it was responsible for termination of protein synthesis at all stop codons. This would imply that the versions of RFH that we see in less than 10% of bacteria are remnants of a decaying gene that is being substituted with more efficient specialized RF1 and RF2. On the contrary, if RFH gene is a product of a recent duplication of one of the modern variants of RF1 or RF2 genes, it could be expected that its function is specific for certain bacterial lineages that share either specific environmental conditions or certain aspects of metabolism (similarly to distribution of Pyl-insertion machinery among methanogenic organisms [35]). In such a scenario, the existence of a significant proportion of bacteria with *prfH *pseudogenes would be an indicator of unsuccessful horizontal gene transfer events, rather than an indicator of lineage specific gene loss.

**Figure 3:**
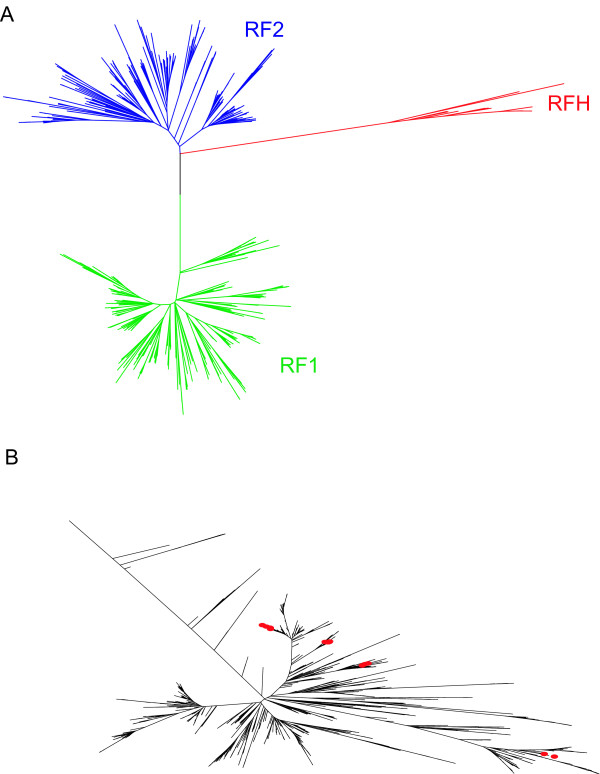
**Phylogenetic tree of bacterial RFs**. **A**. A consensus tree of bacterial RF genes. The tree was constructed with MEGA3 program [36] using neighbor-joining method using a set of nonredundant protein sequences and Dayhof substitution matrix, gaps were deleted during pairwise distance estimations. Branches corresponding to RF1 genes are shown in green, RF2 are in blue and RFH are in red. **B**. Distribution of RFH sequences across the bacterial tree obtained from Ribosomal Database Project 2 [37]. Bacteria in which RFH sequences were found in the present study are marked with red circles. Note that the absence of red circles does not necessarily indicate the absence of RFH sequences.

To attempt to discriminate between different potential evolutionary scenarios for *prfH*, we attempted to perform phylogenetic reconstruction of all bacterial RF genes. For this purpose, sequences of all release factor genes were extracted from completed genomes using ARFA program [22], and an alignment of the corresponding proteins was built using the ClustalW program [23]. The alignment was also backtranslated to produce the corresponding nucleotide sequences [see Additional file 1] (note that one nucleotide in RF2 genes whose expression utilizes ribosomal frameshifting, was removed to make backtranslation possible). To reconstruct phylogenetic trees we used neighbor-joining method and the minimal evolution method implemented in the MEGA3 program [36]. The topologies of trees obtained vary in terms of the location of a node corresponding to RFH origin and depends on the evolution models used and the manner of treating alignment gaps. Both the bootstrap and the interior branch tests indicated a very low level of confidence for the corresponding branches. Therefore, our phylogenetic analysis related to the origin of RFH is inconclusive. However, in the majority of the phylogenetic reconstructions, the node corresponding to RFH divergence is either more close to branches corresponding to RF2 genes or is located within the RF2 sub-tree, suggesting that RFH is evolutionarily closer related to RF2 than to RF1. A consensus tree obtained by the neighbor-joining method and Dayhoff matrix as a substitution model, is illustrated in Figure 3A. Detailed information on a tree shown in Figure 3A can be found in the additional file that can be viewed with MEGA [see Additional file 2].

It is hard to estimate the contribution of horizontal gene transfer to the evolution of RFH. Fig. 3B shows the distribution of RFH genes in the bacterial phylogenetic tree (based on sequences of small ribosomal subunit rDNAs) obtained from the Ribosomal Database Project 2 [37]. Branches corresponding to bacteria where RFH genes were found are indicated by red circles. Note, that the absence of red circles does not indicate the absence of RFH genes in the corresponding bacteria, since the complete genome sequences of many bacteria represented on this tree are not available. It is clear that RFH occurs in distantly related bacteria. It is possible that horizontal gene transfer contributed to the expansion of RFH across lineages, since most of the bacteria where we found RFH genes are either animal or plant pathogens and therefore can share a common habitat in human guts. In addition, we found one RFH member in marine bacteria and we found a number of RFH encoding sequences (data not shown) in environmental samples obtained from the Sargasso Sea [38]. Again, this does not preclude a possibility of horizontal gene transfer, given human preferences for the sources of nutritional supplements and waste management.

We also believe that a more extensive analysis of RFH origin is necessary to obtain the most likely evolutionary scenario of RFH, but it is outside the scope of the current study. Reconstruction of true phylogeny for release factors is a complex problem, since it is likely that homologous recombination between paralogs has contributed to the evolution of corresponding RF genes due to the high level of their sequence similarity at certain conserved sites. A likely example of such homologous recombination can be seen in the alignment in Fig. 1A for the insertion common to both RF1 and RF2 sequences from *Bacteroides thetaiotaomicron*.

## Testing the hypothesis

The essential step towards testing our hypothesis is the reconstitution of an active RFH (if none of the present *prfH *genes encodes an active variant). There is a high degree of conservation of particular features in RFH, which suggests that an active form can be restored. Yet, in *E. coli *K12 and many other bacteria, the RFH gene is present as a pseudogene. Disruptions in the corresponding ORFs are illustrated in Fig. 2A. It is also possible that some genes contain inactivating sense mutations. It is particularly hard to reconstitute an active form of the protein and assay its activity when its function is not fully understood. It is unclear what kinds of signals are recognized by RFH. Thus, the first step needs to be the identification of the mRNA signal that it recognizes. In this regard, the present knowledge of the medium resolution *in situ *cryo-EM and crystal structures [9,16-18] and high resolution isolated crystal structures, of RF1 and RF2 [7,8] are very helpful. We suggest that residues in the anticodon loop and in the tip of the alpha 5 helix in RF1 and RF2 should be substituted with those from RFH, or perhaps more significant swapping of larger parts of domains should be pursued, and then selectivity towards mRNA should be assayed in the absence and in the presence of the co-conserved upstream gene product. For example, a change of G (in RF1 and RF2) in position 138 (boxed in Fig 1A) to P (in RFH) should not change the selectivity of the RFs towards positions 2 and three of the stop codon, but may change the selectivity towards the first position. A set of such experiments with partial and complete swaps of residues in RFs interacting with mRNA may reveal an alternative signal recognized by RFH. Mora et al [13] changed specificity between RF1 and RF2 by swapping 16 residues in the mRNA recognition domain using such a strategy. Despite the seeming simplicity of such experiments, the elucidation of RFH signal may not be straightforward. Possibly the design of a chimeric RF, like the one used in the Ito et al study [12] would be needed. After a potential RFH signal is found, it will become possible to test naturally encoded RFH for activity as class-I RFs, and subsequently screen for a function of the co-conserved upstream gene.

Alternatively, elucidation of the function of the upstream gene product may point towards potential RFH signals.

## Implications of the hypothesis

The evolution of known well established class-I RFs itself holds several unsolved puzzles. Since there is no strong evidence for an evolutionary relationship between bacterial class-I RFs and their counterparts from archaea and eukaryotes, it is unknown how termination was mediated in the last common ancestor. If there was an RNA-based factor similar to tRNAs, was it independently substituted with convergently evolved protein analogs after the kingdoms of life split? It is unknown why there are two class-I RFs in bacteria, while for most organisms from the other kingdoms one factor serves the purpose well. Even among bacteria themselves, there is a small group of *Mycoplasma *and *Ureplasma *species which have lost their RF2 genes (UGA was reassigned to encode Trp). These bacteria rely on a sole RF1 for recognition of their remaining stop codons. Yet these are obligatory pathogens with highly reduced genomes, and no free-living bacterium is known to lack either RF1 or RF2. Presumably, strong selective pressure preserves two class-I RFs in bacteria, although the benefits of having two factors with overlapping specificity are not apparent.

The hypothesis presented here of a third class-I RF does not simplify the situation. On the contrary, it makes it seem even more complicated. Nevertheless, even though experimental investigation of RFH may not give simple answers to above questions, it will help to recreate a more accurate picture of RF evolution. The most provocative aspect of the RFH story is the lack of an apparent need for yet another class-I RF. It is unclear what kind of signals RFH might recognize in mRNA.

Specific and conserved alterations (compared to RF1 and RF2) in those parts of RFH that interact with mRNA suggest that RFH recognizes something different from normal stop codons. Several speculative suggestions can be made regarding what might be a potential RFH signal. We will mention a few of them. If RFH recognizes a combination of standard nucleotides in mRNA other than stop codons (specifically or non-specifically), it will compete with tRNAs. This will result in ambiguous translation of sense codons as stop codons. Under normal conditions, such ambiguous translation is unlikely to be beneficial. However, during starvation for certain amino acids, premature termination on their corresponding codons will release stalled ribosomes. Hence, such a situation might be beneficial if RFH is expressed under starvation conditions for one or more amino acids. This would be useful in dealing with the ribosomes whose A-site is unoccupied in contrast to the RelA mediated stringent response triggered by stalled ribosomes occupied with deacylated tRNAs [39]. Since equilibrium between such ribosomal states is likely, RFH may act with RelA in parallel. If correct the function of RFH would partially overlap with that of tmRNA, but it would not have the tmRNA feature of ensuring the addition of a C-terminal tag, which is the substrate for a specific protease that rapidly degrades the product.

The co-occurrence of RFH and the upstream gene, may also represent a toxin/antidote balance. Unwanted premature termination (performed by RFH) would be toxic, and should be closely controlled by another protein, here suggested to be the upstream gene product.

Another potential role for RFH could be in recognition of mRNA containing nucleotides that are modified because of damage or for other reasons. The list of potential signals could be continued. Whatever the RFH function is, RFH is dispensable in most modern bacteria, meaning that either its function is also dispensable or it is accomplished by a different parallel system.

We know other examples of organisms with additional RFs. In *A. thaliana*, there are three highly similar isogenic eRF1s [40]. In some ciliates, e. g. *Euplotes *and in certain methanogenic archaea, there are two class-I RFs instead of only one [41,42]. Interestingly, in the genetic codes of ciliates and methanogenic archaea, stop codons have been reassigned to sense codons. In many *Euplotes *UGA is reassigned to tryptothan [41], while in methagenic archaea UAG is translated as pyrrolysine [43]. The corresponding RF1s in these species have multiple substitutions in the area of the NIKS motif that is responsible for stop codon discrimination [42]. Whether the emergence of RFH was a result of a similar codon reassignment event is another interesting question to be answered.

## List of abbreviations

tRNA, transporter ribonucleic acid; mRNA, messenger ribonucleic acid; RF, Release Factor; GTPase, guanine triphosphatase; prfH, protein RF homologue; PDB, protein data bank; ORF, open reading frame; PTC, peptidyl transferase center.

## Authors' contributions

PVB conceived and developed the hypothesis, analyzed bacterial genomes for the presence of RF genes, performed comparative sequence and phylogenetic analyses, and jointly wrote the manuscript. BV developed the hypothesis, modeled RFH tertiary structure, performed comparative structural analysis and jointly wrote the manuscript. TH performed initial phylogenetic analysis of genes encoding class-I RFs and contributed to writing and revision of the manuscript. RFG and JN critically evaluated the hypothesis and revised the manuscript. JFA encouraged and developed the hypothesis, assembled the initial team of researchers and significantly contributed to writing and revision of the manuscript.

## Reviewers' comments

***Authors' note: ***The original version of the manuscript (prior to the revision) referred to the product of *prfH *as to RF0. In the revised version we substituted RF0 with RFH as two referees suggested. Nevertheless, some of reviewers' reports use the term RF0 and we have left it as is for clarity. We would like to advice readers that both terms, RF0 and RFH, refer to the same protein product.

### Reviewer's report 1

Daniel Wilson, AG Ribosomen, Max-Planck Institute for Molecular Biology, Berlin, Germany (nominated by Eugene Koonin, National Center for Biotechnology Information, National Library of Medicine, National Institute of Health, Bethesda, MD USA)

The manuscript of Baranov *et al*. hypothesizes that in addition to the usual two class-I release factors RF1 and RF2, there are some bacteria that contain a third class-I release factor, termed here RF0. The manuscript expands on the observation of Pel and coworkers that the *E. coli *strain K12 had a gene that exhibited high similarity to the canonical release factors RF1 and RF2 and therefore termed the protein factor RF-H (release factor homologue) and the gene *prfH*. In light of the many fully and partially sequenced bacterial genomes, Baranov *et al*. reveal that the *prfH *gene is found in only 10% and that the bacteria are phylogenetically distinct and from different environments. In many cases the *prfH *gene is not intact, containing deletions or truncations, suggesting it is a pseudogene, at least in these organisms. Interestingly, an ORF directly 5' to the *prfH *gene is found to be conserved in all cases, whereas an ORF with similar conservation is not found in *prfH *lacking organisms, and the stop codon of the upstream ORF overlaps with the start codon of *prfH *suggesting translational coupling. The expected protein product from the *prfH *gene, if expressed, would be a minimal RF in that it lacked domain I. The conservation of the GGQ motif suggests that this factor would be able to hydrolyze the polypeptide from the tRNA, whereas slight deviations from RF1 and RF2 in the regions approaching the mRNA codon, leading to the suggestion that specificity of the RF0 would be distinct from the canonical termination factors.

Clearly, the fact that in some cases the *prfH *is a pseudogene suggests that it is not an essential factor (at least in these organisms), however the appearance of this gene in unrelated bacteria, the possibility of translational coupling with a mysterious upstream gene and the altered but conserved codon recognition elements, combine to produce an intriguing situation that warrants further investigation. Publication of this hypothesis in *Biology Direct *should bring this mystery to the attention of the relevant researchers capable of pursing this problem.

Some minor points to consider for revision:

1. In the Background section, the *E. coli *K12 *prfH *gene is referred to as a pseudogene. This may well be the case, especially considering that compared to the intact *prfH *genes it has a rather large deletion. But to my knowledge the expression from this gene has not been checked and it may not require the upstream gene. Therefore "likely or probably pseudogene" may be a more cautious term? Furthermore, the way this paragraph is currently written, it implies that Pel *et al*. 1992 have termed it a pseudogene but Pel *et al*. actually suggest that RF-H may be expressed and identify a 141 codon ORF starting with AUG and ending with UAA. However, this said, it should be pointed out that in support of the pseudogene idea is the peculiarities of this strain. Namely, that it has been suggested to have undergone heavy mutagensis, which was used as a possible explanation why RF2 in K12 strains contain Thr246 (making it inactive upon overexpression), instead of canonical Ala like other *E. coli *strains (see Dincbas-Renqvist *et al*., (2000) *EMBO J *and references therein).

***Authors' response: ****This is correct. Pel et al identified E. coli K12 sequence homologues to release factors. Correspondingly, we changed "pseudogene" to "genomic element encoding a protein sequence" in the revised version of the manuscript. The notion that E. coli K12 prfH is a pseudogene was made during our study. Our conclusion on E. coli K12 prfH as a pseudogene is not based on expression data, but on comparison of the prfH sequence from E. coli K12 with other prfH sequences. For example when prfH and its upstream gene from E. coli K12 are compared with E. coli CFT073, there is a deletion of about 1,000 nucleotides *(*see *Figure 2A), *but other than that, their sequences are nearly identical. This thousand nucleotides deletion encompasses the stop codon for the upstream gene and the start codon for prfH, plus large bulks of both protein sequences. Even if the corresponding region of E. coli K12 genome is transcribed under certain conditions, its translation would be significantly impaired. Even if some protein products would result from such translation, they cannot have the same function as proteins encoded by intact genes. The translation product would lack one of the central β-strands of the β-sheet in domain 2, thus the domain structure would be heavily altered, if at all folded. Therefore, prfH and its upstream gene in E. coli K12 may be referred as pseudogenes according to the pseudogene.org definition: "Pseudogenes are genomic DNA sequences similar to normal genes but non-functional; they are regarded as defunct relatives of functional genes." Though, clearly this definition can be interpreted differently, since the terms 'function' and 'gene' are not entirely unambiguous. Besides this definition seems to be imprecise at least because pseudogenes are not necessarily limited to DNA entities*.

2. Background section, para 2: while it is true that "Structural studies revealed that such RF anticodons are in close proximity (if not in direct contact) to positions 2 and 3 of stop codons", it might be appropriate to cite some of the biochemical data that first revealed this, for example, the crosslinking data from the Tate lab (Brown and Tate, *JBC *1994; Poole *et al*.,*RNA *1997).

***Authors' response: ****We agree. We included corresponding references and appropriate text in the revised version of the manuscript*.

3. In the Hypothesis section, it would be nice to have the exact number of RF0 containing genomes, with the division of those that are intact and disabled, as well as perhaps what sort of deletions there are. If Figure 2A shows all the RF0 genes that have deletions then this should be stated.

***Authors' response: ****While this manuscript was under review, we have developed a computer program ARFA (Automated Release Factor Annotation) which is available at *[46]. *A manuscript describing ARFA was recently published in Bioinformatics, see *[22]. *While the primary goal of ARFA is annotation of programmed ribosomal frameshifting in genes encoding bacterial RF2, it also discriminates between RF1, RF2 and RFH. Analysis of 311 completed bacterial genomes available at RefSeq on 20*^*th *^*of May revealed 23 genomes containing prfH genes or pseudogenes. We have updated the revised version of the manuscript with this information. Obvious inactivating mutations (large deletions, frame shifts and nonsense mutations) are illustrated on *Figure 2A* and described in the corresponding text. We cannot exclude the possibility that certain amino acid substitutions can result in deactivation of these genes and, therefore cannot give a precise prediction of how many genes are disabled*.

4. Hypothesis section, para 2. Since the *yaeJ *gene is mentioned here, I think it should be briefly described, otherwise the reader is left feeling ignorant.

***Authors' response: ****yaeJ is another bacterial gene with a conserved GGQ motif. Since other parts of yaeJ do not share significant sequence similarity with RFs, it is unlikely that yaeJ functions as an RF. We gave an appropriate brief description in the text*.

5. Hypothesis section, para 2: "All together, these combined differences suggest different codon specificity for RFH." Either that or they suggest non-functionality!! Similarly in the 'Implications of the hypothesis' section, para 3: "Specific and conserved alterations (compared to RF1 and RF2) in those parts of RF0 that interact with mRNA suggest that RF0 recognizes something different than normal stop codons." May also simply reflect inactivity!!

***Authors' response: ****There are usually many ways to break or inactivate something. We see this also with the example of the prfH gene here, where different obviously disabling alterations can be found. However, the RFH "tripeptide anticodon" is conserved among all prfH genes and their alignment points to evolutionary selection of corresponding residues. Therefore, we believe that corresponding alterations in RFH have a functional meaning. This notion is a foundation of our hypothesis that the prfH product evolved to recognize a specific mRNA signal, which is different from those recognized by RF1 and RF2. If the RFH "peptide anticodon" were non-functional, we would expect many more variants of it than just SXY*.

*Nevertheless, without experimental evidence we cannot exclude a possibility that a conservation of this motif in RFH is due to other constrains than specificity in mRNA recognition*.

6. Background, para 2: domain I of RF0 is missing. As mentioned, this domain is not essential and is probably involved in recycling through interaction with RF3. I think the fact that RF3 itself is not essential and even missing in some organisms should also be mentioned here since this is in line with the dispensability of domain I.

***Authors' response: ****This is a very good point. We added this information and relevant references to the revised manuscript*.

7. 'Implications of the hypothesis' section, para 2. Perhaps "truer" should be replaced with "more complete"?

***Authors' response: ****Corrected, it is now "more accurate"*.

8. 'Implications of the hypothesis' section, para 3. One scenario that the authors raise for the function of RF0 is releasing of stalled ribosomes during conditions of amino acid starvation. Although not mutually exclusive, it should be recognized that under such conditions the uncharged tRNA binds at the A site and would prevent RF0 binding. It is the binding of the deacylated tRNA that triggers the RelA-mediated ppGpp synthesis that characterizes the stringent response (see Wendrich *et al *(2002) *Mol Cell *and references therein).

***Authors' response: ****We agree with this point. If prfH plays a role during starvation, its role will be relevant to RelA-mediated stringent response. However, prfH function is not necessarily similar to the one of RelA. RelA is responsible for global changes in the gene expression at the transcriptional level and it binds to ribosomes whose A-site is occupied by deacylated tRNAs. The prfH product would be responsible only for the release of the stalled ribosomes whose A-sites are empty. These two activities would be needed for the different purposes (ribosome rescue vs. stringent response) and may occur at different conditions. To our knowledge, it is not known precisely what proportion of the stalled ribosomes is occupied with deacylated tRNAs compared to stalled ribosomes with empty A-sites. Likely, there is an equilibrium between these two states, since deacylated tRNAs are bound to the ribosome reversibly. We made minimal changes to the text to expand discussion of this particular hypothetical prfH function in relation to RelA-mediated stringent response*.

9. The appearance of the *prfH *gene in unrelated bacteria is not thoroughly discussed in evolutionary terms i.e. horizontal transfer versus gene loss etc. The "anticodon" motif of RFH (SXY) is similar to RF2 (SP(F/Y)). Can it be said if RF0 is more closely related to RF2 than RF1? i.e. did it arise from duplication of the RF1 gene or are they equally related such that RF0 may be progenitor to both RF1 and RF2 genes and simply has been lost in some organisms.

***Authors' response: ****This comment is similar to the second comment of reviewer 4 (Eugene Koonin) We performed additional phylogenetic analysis of RFs encoded by completed sequenced bacterial genomes. We discuss possible RFH evolutionary scenarios in the text of the revised manuscript in detail. Based on the analysis we think that the similarity between RFH and RF2 "anticodon" motifs in part can be explained by closer relationship of RFH to RF2 than to RF1. This does not necessarily mean that RFH selectivity to mRNA is more similar to the one of RF2 than the one of RF1*.

*Also, see our response to reviewer 4*.

10. Lastly, I am not sure that RF-H warrants renaming just yet. I think if it is demonstrated to have release factor activity, then RF0 may be an appropriate name, depending on what its function turns out to be. However, at the moment I think RF-H, release factor homologue, is perhaps a more careful description.

***Authors' response: ****This comment is parallel to the one by Reviewer 3 (Warren Tate). We believe that a consistency among referees is an indicator of the virtue of this suggestion. We have removed the naming RF0 in the revised version and substituted it with RFH*.

### Reviewer's report 2

Yoshikazu Nakamura, Department of Basic Medical Sciences, Institute of Medical Science, University of Tokyo, Japan (nominated by Eugene Koonin, National Center for Biotechnology Information, National Library of Medicine, National Institute of Health, Bethesda, MD USA)

The manuscript by Baranov *et al*. proposes a provocative, though yet unidentified, function of a novel member, referred to as RF0, of class-I release factor in bacteria. The RF0 sequence was first reported some fifteen years ago by Pell and colleagues in *E. coli *K12 genome as a pseudogene that shares significant sequence conservation with two functional release factors RF1 and RF2. Since then, regardless of having attracted strong interest in this field, no significant progress has been made. To my knowledge, this is the first most comprehensive characterization of RF0 based on the available sequence database coupled with the 3D structural modeling. Based on the highly conservative nature of RF0, they propose that RF0 should possess, or should have possessed, the decoding function in translation. This prediction immediately generates several interesting questions. Why RF0 is pseudogene and silent? Does it potentially recognize a specific codon or not? Is there any circumstance to activate or express RF0? etc. etc. The authors elaborate their prediction and working hypothesis in a theoretical way. I found this manuscript is quite interesting and deserves publication in *Biology Direct*. I trust that the following comments might be useful to revise the paper.

1. I am not clear if RF0 is not expressed in ANY organisms or not. This point must be clarified from available information in the literature or "data not shown" information if available.

***Authors' response: ****We have not found any published evidence of RFH expression in any bacteria. Therefore, we can state that it is currently not known whether RFH is expressed in any bacteria under any conditions. Such a statement is added to the revised version of the manuscript*.

2. The putative RFH anticodon "SXY" seems to be a SPF (RF2) type. Nevertheless, "Y" has never been appeared in our previous extensive selection (Nakamura and Ito, *FEBS Letter *514: 30–33, 2002). Hence, I feel it may not be a RF2 or omnipotent type – something different. This might be useful to your argument.

***Authors' response: ****Yes, indeed, in previous studies phenylalanine was always found in the third position of RF2 "peptide anticodon". However, in this and in our other recent study *[22]* we found a small number of RF2s with tyrosine at this position. It is not possible without experiments to determine whether this amino acid substitution will alter the specificity of the RFs in question. However, both amino acids are bulky aromatics, thus it is likely that the specificity of such RF2s is unaltered. Therefore, we referred to the RF2 peptide anticodon as to SP(F/Y). On the other hand, phenylalanine is clearly predominant at this position among all RF2s, while tyrosine is almost universal (with one exception where tyrosine has been substituted by a tryptophan) at the corresponding position in RFH. Similarly, while proline is predominant in the second position of RFH "peptide anticodon", it is universal in RF2 at the same position*.

*We agree that this can be interpreted as an indicator of different specificity, but we cannot estimate the depth of this difference*.

3. Although the above possibility of RF0 reading some sense codon(s) is fascinating, they might take another possibility into consideration as well. That is, loss of specificity of reading. It is known that charge-flip variant RF2 proteins, altered at conserved Glu residues adjacent to the SPF motif, trigger polypeptide release at non-cognate stop, and even sense, codons (Ito *et al*., 1998; Uno *et al*., 2002). These Glu residues are exposed on one side of the surface of domain 2/4 of RF2, suggesting that electrostatic interactions between a class 1 RF and the ribosome are important for the accurate docking in the ribosome (Nakamura and Ito, 2003). Therefore, given some circumstance allows to express RF0 in urgent conditions, it is likely that RF0 functions to stop translation at any codons. It is interesting to speculate this as a novel rescue system.

***Authors' response: ****Indeed the residue attached to the "peptide anticodon" is usually negative (with a few exceptions). On the contrary, in RFH there is a conserved positive arginine. We agree that it is very likely that this change contributes to mRNA specificity of RFH and indicates that this specificity is different from RF1 and RF2 and now mention this fact in the revised manuscript*.

*Although we believe that the high conservation of the amino acid motif in the area of the "peptide anticodon" indicates specificity, we cannot exclude that there might be other reasons behind such conservation and RFH binds to mRNA nonspecifically. It is also possible that RFH will function to rescue stalled ribosomes*.

4. Finally, I am not so confident that simple transplantation of RF0 anticodon "SXY" into the RF2 sequence does work. Rather, as shown in our paper (Ito et al. Nature 2000), a chimeric RF1/RF2 construct might be useful for the anticodon swap experiment.

***Authors' response: ****We absolutely agree and these were our original intentions. One example would be to also make a shorter 'peptide anticodon' loop. We now describe this part in greater detail for clarity*.

### Reviewer's report 3

Warren Tate, Department of Biochemistry, University of Otago, Dunedin, New Zealand (nominated by Eugene Koonin, National Center for Biotechnology Information, National Library of Medicine, National Institute of Health, Bethesda, MD USA)

Summary: This manuscript highlights an interesting and intriguing question about the role of a prokaryotic release factor (RF) orthologue (RFH) that has the key tripeptide motifs for codon recognition, and for peptide release but lacks domain 1 of the classical class-I RFs and has no known function. This means it has the potential to recognize signals in mRNA and contact the peptidyl transferase centre of the ribosome but may form a different kind of interaction with the ribosome. The potential function of these proteins is intriguing: on the one hand, the gene appears to be non functional in some species by appearing as a pseudo gene, whereas it appears to be complete in others. The fact that it sits beside remnants of an ancestral gene cluster encoding a novel flagellar system in *E. coli *K12 (Ren *et al*., *J Bacteriol *2005) is intriguing since it invokes the thought RFH could have been associated with a specific case of termination in the past (rather like SELB vs EFTu in elongation). It is also intriguing that the initiation factor, IF1, family has the decoding domain of the RFs and the tripeptide motif of RFH is more similar to this although IF1s lack a GGQ. These proteins have the OB fold (IF1) (or partial OB fold in the case of the RFs), and presumably RFH has it also.

***Authors' response: ****We highly appreciate these comments, which are rich in information potentially relevant to the topic of the manuscript. We have decided to use reviewer's pointers and investigate their relation to RFH in more detail*.

*1. Flagellar system, Flag-1 and Flag-2*.

*The Ren et al. article describes a novel enetrobacterial flagellar system that is located in a close proximity to prfH (approximately separated by four-five protein-encoding genes). We investigated whether there is a correlation between occurrences of this flagellar system and prfH*.

*Our brief investigation indicates that there is no direct correlation. These flagellar system clusters is limited to E. coli 042 and other genes associated with this system occur only in certain enterobacteria, while prfH can be found in very distant bacteria. On the other hand, there are enterobacteria containing such flagellar systems but lacking prfH, e. g. Y. pestis. Hence, the connection is not apparent*.

*2. Parallel between selB and prfH*.

*We find this parallel interesting. Let us assume that at some point during evolution, an amino acid containing a rare chemical element was used and this amino acid was incorporated at a specific codon. In this, case, termination of translation at such codon will be beneficial when bacteria are placed in a habitat lacking this specific element. Hence, a special termination factor that is expressed under certain conditions would be needed*.

*3. Decoding domain of IF1*.

*We understand that by decoding domain in IF1, the referee means the site of IF1 that binds to the ribosome close to the mRNA location. Indeed, IF1 and RFs have somewhat a similar fold and there is a tripeptide (TPY in E. coli and SPY in some other bacteria) which may interact with mRNA. This, said, it is unlikely that IF1 recognizes mRNA in a manner similar to RFs and to RFH in particular. At least there is no reason to believe that IF1 recognizes mRNA in a specific manner. The existence of TPY in IF1 and SXY in RFH in the loops assumingly interacting with mRNA is intriguing. This may reflect the observation that also the 'peptide anticodon' of RFs seemingly interacts with both rRNA and mRNA, thus the rRNA binding site of the motif could be similar for IF1. Yet, we believe that making speculations based on this observation would be too far-reaching. Hence, we did not modify our manuscript, a curious mind will be able to read referees comments and will find it here*.

The manuscript provides some provocative ideas as to what RFH might be doing and some suggestions for experiments to test whether it recognizes a different stop signal, perhaps differing the first base. This are readily assessable although our ideas of recognition might still be too simplistic, despite the compelling modeling of the X ray derived densities of the decoding RFs loops in a termination complex at the decoding site (Petry *et al*.,*Cell *2005). GG (or GP) motifs in both RF domains indicate sharp turns in the structure marking the extremities of the loops that may relate to the functions but may not be an integral part of them-this is still to be determined but is an important question to resolve.

***Authors' response: ****We agree that GG/GP conservation may not be directly related to selectivity of the first position in stop codons and mention this in the manuscript now. Yet, we believe, that GG/GP are the best candidates as residues that are responsible for the first stop codon position discrimination, since there is no other universally conserved residues in this area*.

My own view of the proposed name RF0 is that locking the nomenclature of this group of genes too closely to the existing families of RFs (RF1 and RF2) at this stage might be premature when we do not know whether they function to recognise stop signals or have a release function in termination. Hence, I would prefer a name like RF-like, or even oRF (orthologue of RF) that can be later modified if a closer association with classic RFs emerges with functional data. Nevertheless, the hypotheses are stimulating for those of us involved in experimental testing of the importance of residues and motifs in the RF families. This is a very worthy contribution to the discussion and intellectual argument about this group of interesting proteins.

***Authors' response: ****We changed RF0 to RFH, see our response to a similar suggestion by reviewer 1*.

Some specific comments:

1. The hypothesis is a good one for experimental testing: that is there is another class of RFs that recognise non-conventional signals perhaps in a small number of specific instances.

2. The retention of the two tripeptide motifs that specify codon recognition, and peptide release (the only motif conserved through all RFs) in RFH is compelling although the consequences of the lack of domain 1 are still not totally clear for ribosome function. Domain 1 seems more important for RF2 function than for RF1 (independent of RF3). (This is interesting given that on L11 lacking ribosomes (domain 1 interaction site) RF1 is totally inactive whereas RF2 has several fold higher activity – Tate *et al*.,*J Biol Chem *1984). Domain 1 is called inessential in the manuscript (Background, second paragraph, last line); perhaps the Mora reference could be given with this statement because they were able to show this specifically with *in vitro *assays. This is consistent with our original proposal of the tRNA analogue hypothesis of two essential but conformationally coupled domains, one for codon recognition, and one for release (Moffat and Tate, *J Biol Chem *1994).

***Authors' response: ****We gave a corrected reference to Mora et al*.

3. The gaps, GP, and additional amino acid (172) in the anticodon loops of RFH suggest it will be important to determine what flexibility there is in this region before losing codon recognition capacity.

4. It is interesting that the RFHs have the IF1-like SPY (203–206), and lack RF type sheet structures around that feature ie following the conserved G (195) and following (~210+). IF1, RFH, and the conventional RFs look like a family of proteins with loops that have specific base interactions.

***Authors' response: ****Yes, changes in the vicinity of the SXY motif strongly suggest that its mRNA specificity is different from RF2, despite some similarity to its "RF anticodon" SP(F/Y). However, we can predict neither exact folding of the corresponding loop, nor its precise effect on mRNA recognition*.

5. The discussion of the implications for the evolution of a protein decoding mechanism for stop codons is particularly interesting. If this were originally non-specific or RNA mediated then an existing protein might have been captured for this purpose. Did a protein like RFH carry out a specific function (accelerated release of a protein from the ribosome?) that was generalised with the development of the RF1 and RF2 families and the acquisition of domain 1 and RF3 functions? As asked by the authors, why have three families of decoding factors, or even the well documented two families. Relevant to this is that the RF2 family has the conserved frameshifting mechanism associated with its expression whereas the RF1 family does not. There are a number of unresolved questions.

***Authors' response: ****We agree with the referee and believe that this comment does not require any changes in the manuscript*.

6. We have expressed the K12 version, while realizing it had a shortened N terminus – it expressed well so was not toxic but all ended up in an inclusion body (perhaps the reason for lack of toxicity). Our next attempt is to use the sequence in *E. coli *042 (Ren *et al*.,*J Bacteriol*. 187 (Feb2005) p1430 where the ancestral 44 gene cluster (Flag2) abuts prfH (are they connected?).

***Authors' response: ****The completed genome of E. coli strain 042 was not available at NCBI when we made the most recent analysis and in fact it is still not available (referred as in progress at the moment when these words are written – July 7*^*th*^, *2006). Hence, it is not included in our analysis*.

*However, the sequence of E. coli 042 genome is available at the Sanger center*.

*Just for the purpose of this comment, we performed BLAST analysis of prfH and its upstream gene from E. coli strain CFT073 against E. coli strain 042. Nucleotide sequences. of prfH and upstream gene from E. coli 042 are 96% identical with no indels in the produced alignment*.

*On the contrary, the 44 gene cluster described in Ren et al article is unique to E. coli 042*.

*According to our diminutive analysis, E. coli strains CFT073, 042 and UTI89 contain active (at least uninterrupted prfH and upstream genes). E. coli strains K12 MG1655, O157:H7 str. Sakai, O157:H7 EDL933 and W3110 contain ~1000 nts deletion in the area covering C-terminal part of upstream gene product and N-terminal part of RFH*.

7. The lack of a domain 1 is interesting – does that suggest it has a more transitory association with the ribosome as well as the lack of an RF3 interaction site, or perhaps a blocking role like IF1 in the A site during initiation? If there are two binding states (as we believe) the first dependent upon domain 1 and then, on correct codon recognition a second state perhaps involving correct positioning of the GGQ (completing unfolding of domain III)-then can RFH go into the second state without a docking at L7/L12/L11. There are the reports now suggesting the RFs can function without their domain 1 (RF-1 in particular *in vitro *and both *in vivo *but with slow growth). These are intriguing question thrown up by the ideas expressed in this manuscript.

***Authors' response: ****We agree that the lack of a domain 1 is intriguing, and may also be significant in suggesting a potential role of RFH*.

*a) It is possible that domain 1 did not exist in the common progenitor of bacterial RFs. The class II RF RF3 enhances class 1 RF function on the ribosome due to recycling, which assumingly happens via domain 1. But as the reviewer mentions, domain 1 is not essential for function. Domain 1 is also the least conserved domain in RFs. It has further been shown that domain 1 is flexible in solution, which further adds to the appearance as an 'added' domain*.

*b) Indeed the lack of an efficient RF3 mediated recycling of RFH must be a consequence of the lacking domain 1. However, spontaneous dissociation of RFs from the ribosome does happen (as proven in vitro and in vivo) thus RFH would perhaps not block ribosomes, but cause slow recycling. However, given the further differences in RFH sequence, the RFH binding to the ribosome may also be either strengthened or weakened. In our opinion, it is difficult to judge the effect of the absence of domain 1 on the speed of dissociation, since that also depends on the affinity of RFH towards ribosomal binding site, but it will preclude interaction of RFH with RF3*.

### Reviewer's report 4

Eugene Koonin, National Center for Biotechnology Information, National Library of Medicine, National Institute of Health, Bethesda, MD USA

This paper presents a simple and straightforward hypothesis regarding the function of the bacterial PrfH proteins, a homolog of class 1 release factors. It is proposed that PrfH is RF0, a novel release factor with a distinct specificity. Of course, it is hard to disagree with this prediction – given the high level of similarity between PrfH and experimentally characterized release factors. I may note that, in the COG database that is used in this paper, and in other databases, PrfH proteins are annotated as putative release factors, so realistically, the novelty of the hypothesis is not so dramatic. Of course, a detailed discussion of potential functions of these uncharacterized proteins is useful. Herein, however, lie some problems with the current version. Again, given the rather obvious nature of the main idea, the value of the paper is expected to be in detailed analysis, and this seems to be somewhat underdeveloped. Specifically, I see three rather substantial issues:

1. Unfortunately, the manuscript includes no prediction of the signal recognized by RFH. This is understandable as there is, apparently, not enough data for making such a prediction. This being the case, however, I feel that the title of the paper is somewhat misleading because "...atypical mRNA signals, other than normal stop codons" seems to imply a specific prediction (the discussion of some possibilities at the end of the paper is really vague). For that matter, I am not convinced that this aspect of the hypothesis holds once the experiments are done: it is quite a possibility that RF0 does recognize one or more of the standard stop codons but under some specific conditions.

***Authors' response: ****Our hypothesis has two components:*

*1. RFH is a class-I release factor*.

*2. RFH mRNA recognition is different from RF1 and RF2*.

*The novelty of the first component indeed is not dramatic. In their original work, Pel and colleagues also hypothesized that prfH gene encodes a release factor homolog. Because of a very high sequence similarity, it is not surprising that databases refer to corresponding genes as encoding putative release factors. Moreover, in several cases prfH genes are annotated as putative RF2 genes in the RefSeq database (see ref. 22)*.

*The need of an article about prfH is easy to illustrate. If someone will query Medline for the term prfH, no results will be returned. Based on our analysis, this gene certainly deserves more attention. Further, it is an important point, that the original finding by Pel et al *[20]* concerned a gene that probably does not have a functional protein product. The deletion of amino acids encoding a central β-strand in the β-sheet in domain 2 is so dramatic that the protein unlikely folds. Thus, the report of potentially folded and perhaps functional gene products in evolutionarily distant bacteria, still represents novelty. However, the major critical point of the referee seems to be not a lack of novelty, rather a lack of sufficient detail. Therefore, we significantly extended our manuscript with additional material*.

*We disagree with the referee's comment regarding the second component of our hypothesis. Although our hypothetical predictions regarding the exact mRNA signals recognized by RFH indeed lack certainty, we do predict (with high confidence) that this signal is different from RF1 and RF2. Although we still do not fully understand how protein-assisted mRNA decoding occurs, we now have a good sense of what protein components of RF are responsible for mRNA recognition. This became possible due to recent progress in genetics and structural studies of release factors. Based on our comparative analysis of the RF areas interacting with mRNA, it is clear that the difference between RFH and other RFs is higher than between RF1 and RF2. The referee believes that RFH is very likely a class-I RF, while he doubts that its mRNA signal recognition is different from RF1 or RF2. In our opinion, both statements are equally hypothetical unless experimentally proven. High sequence similarity points to a common origin, but it does not prove a common specificity or even a common function. Hence, there is a small possibility that RFH is not a class-I release factor, despite very high sequence similarity in the area corresponding to peptidyl-tRNA hydrolysis domain. On the contrary high sequence divergence in the area of mRNA binding domain, strongly suggest functional divergence. Certain comments of the other three referees indicate that they also believe that it is very likely that if RFH is a class I RF, it has a different specificity towards mRNA. Hence, it encourages us to make no change to our original hypothesis regarding RFH mRNA signal*.

2. A phylogenetic tree for Class 1 release factors is presented but potential evolutionary scenarios for RF0 are not discussed. Why is this gene found in only ~10% of the sequenced bacterial genomes? Is it a result of a relatively recent duplication? What was the contribution of horizontal gene transfer to the evolution of this gene and what was the role of lineage-specific gene loss (the importance of the latter is implied by the fact that many bacteria seems to have *prfH *pseudogenes)? I believe all of this deserves explicit and reasonably detailed discussion. Further, it is strange that the tree is mentioned (not really discussed) in the beginning of the section on possible tests of the hypothesis. Does it have anything to do with those tests?

***Authors' response: ****This comment is parallel to one of the comments by referee 1. We are thankful to both referees for the encouragement to perform detailed phylogenetic analysis. We reconstructed possible evolutionary scenarios for RFH, which are now described in the text of the revised manuscript. According to this analysis, there are two most likely scenarios. 1. RFH originated because of a duplication of a common ancestor of RF2 and RFH. 2. RFH is a result of a duplication of one of RF2 genes after their speciation. Irrelative of what scenario is correct it seems that RFH did exist in the bacterial world for a long time and is not a result of a recent duplication. Given the presence of many prfH pseudogenes in modern bacteria (as pointed out by the referee), it seems reasonable to speculate that prfH had a wider distribution among bacteria in the past and its existence in only 10% of modern bacteria is contributed by lineage specific gene loss. Alternatively, prfH genes spread across lineages through horizontal gene transfer and a large number of pseudogenes is evidence of a failure of these genes to find a niche in the metabolism of corresponding bacteria*.

*We agree with the referee that the description of RFH phylogeny does not belong to the "Testing hypothesis" section and we changed its location in the revised version. However, indeed RFH phylogeny is relevant to the "Testing hypothesis" section and we describe the relationship of our evolutionary analysis to potential functional roles of RFH*.

*Our reconstruction of RF phylogeny is inconclusive and clearly, a more focused study is required. We provide additional files as a supportive material for the revised manuscript to ease such a future study*.

3. I am surprised that the authors do not make a bigger deal of (and, essentially, draw no conclusions from) the juxtaposition of the *prfH *gene with *rtcB *and their (reasonably) proposed translational coupling. It is true that the function of RtcB has not been characterized experimentally but it is not an utter mystery. Indeed, the adjacency of the *rtcB *gene to RNA cyclase in a great number of genomes strongly suggest that RtcB is an enzyme of tRNA and/or rRNA processing as briefly discussed in Koonin *et al*. *Genome Biol*. 2004;5(2):R7 (see Table 1 in that paper). The probable coexpression of prfH with these genes is quite intriguing and might hold the key to the actual function of RF0. I am sure this is worth some serious discussion in this paper.

*Authors' response:*

*We agree that indeed the upstream genes that belong to the family of RtcB-like proteins may hold the secret of RFH function. It deserves more attention and we describe it in more detail in the revised version of the manuscript and provide certain additional speculations regarding this gene and its relation to potential function in RNA processing/modification*.

*Moreover, we modified the title of the manuscript to emphasize this point*.

## Supplementary Material

Additional File 1 ClustalW alignment of RF codon sequences in the nexus format. The names of the sequences are given in the following format: RF2_MC_003919.fna – where RF2 – is the name of the factor (either RF1, RF2 or RFH), MC_003919 indicates an accession number (substitute M with N to get an accession number), it also indicates a name of a fasta file from NCBI ftp site which was used in this study, e.g. NC_003919.fna. Note that sequences corresponding to RF2 genes expressed via ribosomal frameshift were modified by deletion of one nucleotide in the frameshift site to correct for ORF disruption. Click here for file

Additional File 2 Saved MEGA3 tree session corresponding to a tree shown in Fig. 3. Names are the same as in the Additional file 1. However, the number of sequences is different, since the trees were reconstructed based on corresponding amino acid alignment and only one member from a group of redundant protein sequences was used for phylogenetic reconstruction. Click here for file
